# Stunting in Nepal: looking back, looking ahead†

**DOI:** 10.1111/mcn.12286

**Published:** 2016-05-17

**Authors:** Madhu Dixit Devkota, Ramesh Kant Adhikari, Senendra Raj Upreti

**Affiliations:** ^1^ Department of Community Medicine and Public Health, Institute of Medicine Tribhuvan University Kathmandhu Nepal; ^2^ Department of Paediatrics Kathmandu Medical College Sinamangal Kathmandu Nepal; ^3^ Ministry of Health and Population Kathmandu Nepal

Nepal has made impressive gains in health and nutrition despite being in a state of political, economic and demographic transition. According to the Nepal Demographic Health Surveys, stunting in Nepal has fallen from 57% in 2001 to 41% in 2011, an annual decline of 1.7 percentage points. A 3.9% annual reduction is, however, required to achieve the global target of 40% fewer stunted children by 2025. The Multiple Indicator Cluster Survey conducted in 2014 by the Central Bureau of Statistics and UNICEF indicated a further reduction in the proportion of stunted children to 37.4%. The recently drafted Sustainable Development Goal for Nepal, 2016–2030 has outlined a daunting challenge of reducing stunting in children under 5 years of age to 1% by 2030 for the long‐term health, wellbeing, human capital development and national economic growth.

Stunting, which is caused by long‐term nutrition deprivation early in a child's life, often begins before birth and is associated with increased risk of mortality from infectious diseases such as diarrhoea, pneumonia and measles in childhood (Pelletier *et al.* 2012; UNICEF 2013). It also leads to irreversible physical and cognitive damage, and poorer educational outcomes later in childhood and adolescence, with economic consequences for the individual, household and community levels (Walker *et al.* 2007). Stunted children who experience rapid weight gain after 2 years of age have increased risk of becoming overweight or obese later in life, with associated higher risk of non‐communicable diseases like coronary heart disease, stroke, hypertension and type II diabetes (Black *et al.* 2013).

The determinants of linear growth failure in Nepal consist of factors operating at different levels of causation and include poverty, low maternal education and food insecurity. Underlying causes include poor caring behaviours, including infant and young child feeding, inadequate access of households to a diverse and quality diet, low access to health care and repeated infections due to unhealthy environment. Less than half (46%) of newborns are breastfed within 1 h after birth, while 70% of infants younger than 6 months are exclusively breastfed. Only 66% are introduced to complementary foods at 6–8 months, and more importantly, complementary feeding is infrequent and inadequate in terms of quality, quantity and safety. Only a quarter of children (24%) are fed with the recommended infant and young child feeding practices (breastfeeding or receiving milk products, four or more food groups, and a minimum meal frequency according to their age and breastfeeding status) (MoHP, NEW ERA, *et al.* 2012).

Almost all stunting takes place in the first 1000 days after conception (Dewey & Vitta 2013). Evidence reinforces the importance of the nutritional status of women at the time of conception and during pregnancy for healthy fetal growth and development (Gluckman & Pinal 2003; Black *et al.* 2013). Intrauterine growth retardation due to maternal undernutrition is known to account for 20% of childhood stunting. Other maternal contributors to child stunting include short stature, short birth spacing and adolescent pregnancy (Prakash *et al.* 2011). Nearly a quarter (23%) of mothers in Nepal give birth before 18 years of age, while about half have given birth by the age of 20. Currently, 18.2% of Nepali women and 25.8% adolescents have a low body mass index. The prevalence of anaemia in pregnant women – despite the intensification effort – stands at 48% and is increasing, which is clearly above the World Health Organisation threshold of ≥40%, indicating that maternal anaemia is a severe public health problem. Thus, the health, nutritional and social status of women is an important area for investment to reduce stunting even further in Nepal.

There exists substantial inequity in stunting rates between population subgroups because of the complex interplay of geographic, social, economic and political realities. Children from the poorest households are more than twice as likely to be stunted (56%) compared with children in the wealthiest households (26%). Similarly, children in rural areas are more likely to be stunted (42%) than those in urban areas (27%), and ecologically, the mountain zone has the highest proportion of stunted children (53%). Past trends show that declines in stunting were greater among wealthier (fourth and fifth) quintiles than the poorest quintile, indicating that an equity lens is important (Bredenkamp *et al.* 2014).

Efforts have recently steered towards scaling up evidence‐based cost effective, nutrition‐specific and sensitive interventions with a focus on reducing undernutrition among adolescent girls, pregnant and lactating women, and all children under 24 months of age. Nepal is an early riser in the global Scaling Up Nutrition movement, and this has substantially elevated and energised the national discourse on maternal and child nutrition. An ambitious Multisector Nutrition Plan, endorsed by the Government of Nepal and supported by Nepal's development partners, is being rolled out. It links undernutrition with water, sanitation and hygiene, social protection, and agriculture and has a focus on the first 1000 days in the poorer segments of society for accelerated reduction of undernutrition by a third within the next 5 years (GoN and NPC 2012). The main emphasis of the plan is to create an organisation at the community level that would assess the nutritional status at the local level, analyse its causes and design programmes to address them. Resources from different sectors, especially health, water, sanitation and hygiene, agriculture, education and local governance, are expected to be utilised to design and implement the programmes. The plan is being rolled out gradually in different parts of the country.

The strong momentum and commitment in the present context must be harnessed with political commitment, policy and programmatic coherence, capacity enhancement and accountability at the national and subnational levels, and community involvement through effective communication and advocacy. Strong monitoring and accountability systems must be put in place to know if current efforts are yielding the benefits that are anticipated.

## Conflicts of interest

The authors declare that they have no conflicts of interest.

## Contributions

MDD developed the draft of the country paper, RKA and SRU reviewed the drafts and provided technical inputs. All authors read and approved the final version of the paper.
